# Loneliness in Breast Cancer Patients with Early Life Adversity: An Investigation of the Effects of Childhood Trauma and Self-Regulation

**DOI:** 10.3390/curroncol30050389

**Published:** 2023-05-18

**Authors:** Rasoul Heshmati, Shahin Azmoodeh, Mina Kheiriabad, Anis Ghasemi, Chris Lo

**Affiliations:** 1 Department of Psychology, Faculty of Education and Psychology, University of Tabriz, Tabriz 5166616471, Iran; 2 Department of Psychology, Faculty of Literature and Humanities, Urmia University, Urmia 5756151818, Iran; 3 Department of Psychiatry, Temerty Faculty of Medicine, University of Toronto, Toronto, ON M5T 1R8, Canada; 4 Social and Behavioural Health Sciences, Dalla Lana School of Public Health, University of Toronto, Toronto, ON M5T 3M7, Canada; 5 Psychology, School of Social and Health Sciences, James Cook University, Singapore 387380, Singapore

**Keywords:** breast cancer, loneliness, childhood trauma, self-regulation

## Abstract

Childhood trauma may be prevalent in the general population, and the psychosocial treatment of patients with cancer may require consideration of the effects of such early adversity on the healing and recovery process. In this study, we investigated the long-term effects of childhood trauma in 133 women diagnosed with breast cancer (mean age 51, SD = 9) who had experienced physical, sexual, or emotional abuse or neglect. We examined their experience of loneliness and its associations with the severity of childhood trauma, ambivalence about emotional expression, and changes in self-concept during the cancer experience. In total, 29% reported experiencing physical or sexual abuse, and 86% reported neglect or emotional abuse. In addition, 35% of the sample reported loneliness of moderately high severity. Loneliness was directly influenced by the severity of childhood trauma and was directly and indirectly influenced by discrepancies in self-concept and emotional ambivalence. In conclusion, we found that childhood trauma was common in breast cancer patients, with 42% of female patients reporting childhood trauma, and that these early experiences continued to exert negative effects on social connection during the illness trajectory. Assessment of childhood adversity may be recommended as part of routine oncology care, and trauma-informed treatment approaches may improve the healing process in patients with breast cancer and a history of childhood maltreatment.

## 1. Introduction

In this study, we investigated the long-term effects of Childhood Trauma (CT) on adaptation to disease later in the life course. Breast cancer is the most commonly diagnosed cancer in women—in 2020, over 2.3 million newly diagnosed cases were confirmed globally [1]. Furthermore, childhood trauma may be prevalent in the general population, with females at greater risk of maltreatment [2], suggesting that psychosocial intervention with breast cancer patients may require special consideration of the effects of childhood adversity on healing and recovery during the cancer experience [3].

There may be multiple social behavioral pathways by which CT may cascade over time to affect mental and physical health in adulthood. For example, in breast cancer patients, CT has been associated with increased psychological distress, poor self-confidence, maladaptive coping skills, persistent feelings of vulnerability, fatigue, depression, and lower quality of life [4]. In this study, we were interested in how CT and the self-regulation of emotion may affect the experience of loneliness and isolation in women with breast cancer.

### 1.1. Social Connectedness as a Conceptual Framework

The experience of social disconnection involving isolation, loneliness, and negative relationships, may be an important part of a risky developmental pathway through which CT contributes to poor outcomes in adulthood [5]. The ability to connect and relate to others may involve contributions from past relational experiences (producing attachment tendencies) in interaction with present relational experiences. A protective developmental pathway of social connectedness would involve earlier positive relational experiences strengthening self-regulation and spurring further development of positive relationships. In addition, social connectedness may buffer against psychological distress across the lifespan, including during the experience of medical illness, which may strain the individual capacity to cope and increase the need for social support. 

We have proposed that the cancer experience may reactivate developmental challenges of the past [6]. The increase in dependency needs and loss of physical functioning may resurface issues concerning the experience of autonomy, the ability to trust others, and one’s worthiness to be loved, key attributes from an Eriksonian perspective. For cancer patients with childhood trauma, these issues may be exacerbated if these self-attributes are less developed because of early adversity. The threat of mortality can trigger the need for life review and a sense of life completion, posing both clinical risk and opportunity if patients wish to re-engage with and make sense of past trauma. Finally, the experience of cancer can involve multiple emotional shocks over time, testing the ability of patients to manage their emotions while maintaining a semblance of their normal relationships amidst an increased sense of personal vulnerability. 

### 1.2. Loneliness

Loneliness is a subjective and potentially pathogenic feeling which happens when a person’s social relationships do not correspond to what is expected of relationships, including perceptions of close others as untrustworthy. Of note in this definition is the experience of being alone despite the presence of social relations. In addition, traumatic childhood events have been associated with loneliness in adulthood, according to social network analyses [7]. 

There may be several pre-disease processes by which loneliness may compromise health-related quality of life, leading to morbidity and mortality. Loneliness may be associated with psychophysiological pathways involving chronic stress and ineffective coping, affecting immune functioning and physiological repair over the long term [8]. In those coping with medical illness, there can also be a reciprocal relationship between psychological and physical well-being, such that unresolved psychological loss may diminish health outcomes, which then further increases psychological suffering. Loneliness in cancer patients may deteriorate quality of life because the lack of meaningful relationships can be demoralizing [9]. Loneliness has been associated with depression, poor immune function, pain, and fatigue in cancer survivors [10]. It can also lead to an increased period of hospitalization and the lack of feeling healed as a turning point in treatment [11]. 

### 1.3. Childhood Trauma

Childhood trauma refers to the experience of emotional, physical, and sexual abuse and neglect. The experience of CT may exert a persistent negative influence on health and well-being across the lifespan [12]. CT may be understood to reduce emotional self-regulation, making it more difficult for individuals to adapt to life adversity [5]. CT survivors may be more vulnerable to psychosocial distress when coping with illness owing to issues with emotional expression and support seeking. Breast cancer patients who suffered traumatic experiences in childhood may have more trouble feeling supported by others and be more prone to develop cancer-related distress. Their choice of coping strategies and health-related behaviors can reduce treatment compliance affecting medical outcomes [13,14,15]. 

### 1.4. Self-Views

Self-discrepancy is defined as the degree of dissimilarity between two aspects of self-knowledge, the ideal self vs. the actual self. The disparity between self-standards and the actual self has received more attention theoretically and experimentally than in applied clinical settings [16]. For breast cancer patients, self-concept may be shifted in at least two ways. First, the body can become a source of fear. Hence, pain and interoceptive sensations that were dismissed may become prominent and disruptive and related to threat. Compulsive body-checking behaviors may be an example of this [17]. Second, self-concept can be reframed as that of a patient or survivor [18]. Therapies (e.g., chemotherapy, radiation therapy, surgery) may cause important physical changes such as breast removal, skin discoloration, hair loss, and sexual dysfunction. Such outcomes lead to modification of self-narrative and bodily self-representation [19]. 

These changes in bodily experience and self-concept may be disturbing and un-integrated, leading to disruptions in personal identity and feelings of difference and change that make social interactions difficult [20]. An injured self-representation in cancer patients can suggest a constellation of mental health management concerns involving views about the future [21], lifestyle and relationships, adherence to treatments [22], and emotion regulation [23]. Meanwhile, greater self-discrepancy has been associated with disruptions in emotion regulation and adaptive control of emotional expression [24]. 

### 1.5. Emotional Expression

Ambivalence over Emotional Expression (AEE) is an inner conflict about wanting to express one’s emotions yet fearing its consequences [25]. Some of the consequences that people may fear include concerns about burdening others or creating conflict, feeling vulnerable or weak, and the risk of rejection. Supportive care in cancer recognizes the importance of psychosocial interventions to promote healthy emotional expression and management, and AEE affects this treatment process [26]. Servaes et al. [27], in their research on breast cancer patients, found that levels of AEE can increase in response to illness. AEE can be considered a reflection of the disease process owing to the magnitude of emotional stakes and not merely a personality trait. The suppression of emotion may lead individuals to suffer alone and in silence [28]. In cancer patients, AEE has been associated with higher levels of pain behavior, being overly reserved or non-communicative, having anxiety and depressive symptoms, poor self-effacing behaviors in relationships, poorer emotional well-being, and non-adherence to treatment [29]. 

### 1.6. Research Aims 

In this study, we were interested in testing a predictive model of loneliness in women with breast cancer as a function of childhood trauma, emotion regulation, and changes in self-concept in the context of illness. In addition to the direct effects of each factor, we examined whether ambivalence about emotional expression may mediate the relationships between childhood trauma and self-discrepant views on loneliness, given that much of the literature would implicate issues of emotional expression as an intervening factor. To our knowledge, this study is the first to examine links between these components directly and indirectly. 

## 2. Method 

### Participants and Procedure 

This study was approved by the Research Ethics Committees of the University of Tabriz (IR.TABRIZU.REC.1401.019). Its design was a cross-sectional survey study of patients with breast cancer and a history of childhood trauma. A research team member approached patients at one private and three public hospitals in Tabriz, Iran, from July to December 2021 and introduced the study to them, asking if they would agree to an initial screening of early trauma experiences. A total of 408 patients with breast cancer agreed to be screened with the Interview for Traumatic Events in Childhood (ITEC) [30], which was administered by two trained clinical psychologists with MA degrees. 

Based on this interview, 172 patients were selected for further study who had experienced at least one of five types of trauma (sexual abuse, physical abuse, emotional abuse, emotional neglect, and physical neglect) in childhood. Additional eligibility criteria included: (a) confirmed clinical diagnosis of breast cancer; (b) receiving usual cancer interventions; (c) between 20 and 70 years of age; and (d) a minimum education of an elementary school degree. 

Patients were excluded if they had: (a) other chronic diseases; (b) a history of alcohol, drug and substance abuse; (c) personality disorders; or (d) severe neurological or auditory impairment. According to these criteria, the eligible sample was reduced to 139 patients. The study objectives were further explained to these patients, and they were invited to provide written informed consent if they wished to participate. All 139 patients consented and subsequently completed the research measures. Among them, 6 participants were omitted due to outlier responses to questionnaires and the final analysis was performed on a sample of 133 participants.

## 3. Measures

### 3.1. Interview for Traumatic Events in Childhood (ITEC) 

The ITEC is a retrospective trauma interview developed by Lobbestael et al. [30]. This tool assesses 5 types of childhood traumatic events, including sexual abuse, physical abuse, emotional abuse, emotional neglect, and physical neglect. The items evaluating sexual trauma are preceded by two screening questions: “Were you ever sexually approached against your will?” and “Did you ever have a sexual relationship with someone who was at least 5 years older?”. The other traumas are not introduced by screening questions. For each category of trauma to which the participants answered positively, follow-up questions are used to collect more information about perpetrators, age of onset, frequency and duration of trauma, and the impact on the victims in the past and the present. The ITEC was used to generate 2 binary variables concerning the experience of physical or sexual abuse (yes or no) and the experience of neglect or emotional abuse (yes or no). 

### 3.2. Child Abuse and Self-Report Scale (CASRS) 

This measure includes 38 items that assess 4 categories of traumatic experience during childhood: sexual, emotional/psychological, physical, and neglect [31]. All items were measured with 5-point Likert scales, anchored by “0 = Never” and “3 = Always”. The neglect subscale (items 15–25) is reverse scored. Total scores range from 0 to 114, with higher scores indicating greater experience of trauma. In this study, this scale had good internal consistency (α = 0.83). To characterize levels of childhood trauma in the sample, we used a cut-off value ≥ 57 to identify cases of moderate severity. We calculated this value by using the scale midpoint and multiplying it by the number of items.

### 3.3. Self-Discrepancies Scale (S-DS) 

This measure assesses discrepancies in self-representation [32]. In this study, participants were asked to generate a list of 12 traits that they would ideally desire to have (positive ideal self) or not to have (negative ideal self). Secondly, they generated a list of 12 traits that others would like them to have (positive ought self) or not to have (negative ought self). For each trait, they scored the extent to which they possessed it on a scale from 0 to 100%. Finally, participants were asked to assess the perceived gap between their ideal and actual selves (ideal gap) and between their prescribed and actual selves (prescribed gap). These items were scored on a 7-point scale ranging from 1 (I feel very close to this ideal) to 7 (I feel very far away from this ideal). They were also asked to rate their distress on two Likert scales ranging from 1 to 7, with higher scores indicating more distress about their ideal and prescribed gaps, respectively. In this study, we took the sum of these four items as an assessment of the overall experience of self-discrepancy (internal consistency = 0.84). This total score could range from 4 to 28, with higher scores indicating greater negative experience of discrepancy. To characterize levels of self-discrepancy, we used a cut-off value ≥ 18 to identify cases of moderate severity. This value was calculated by taking half a point above the scale midpoint and multiplying it by the number of items.

### 3.4. Ambivalence over Emotional Expression Questionnaire (AEQ) 

The Ambivalence over Emotional Expression Questionnaire (AEQ) [25] evaluates wanting to declare feeling to others and being unable to do so or declaring feelings to others and regretting it. The Persian version of the AEQ [33] consists of 23 items (e.g., “I want to tell someone when I love them, but it is difficult to find the right words”) rated on a 5-point scale (1  =  Strongly Disagree, 5  =  Strongly Agree). The total score ranges from 23 to 115, with higher scores indicating greater severity of ambivalence. This questionnaire showed adequate internal consistency here (α = 0.72). To characterize levels of emotional ambivalence, we used a cut-off value ≥ 80.5 to identify cases of moderate severity. This value was calculated by taking half a point above the scale midpoint and multiplying it by the number of items.

### 3.5. UCLA Loneliness Scale-Version 3 (UCLA LS3)

This is a one-factor measure that assesses the subjective feelings of loneliness and social isolation [34]. The UCLA LS3 has been developed and revised from the original version, consisting of 11 negative items (e.g., “How often do you feel that your relationships with others are not meaningful”) and 9 positive items (e.g., “How often do you feel that you can find companionship when you want it”). The items are rated on a 4-point Likert scale from 1 (never) to 4 (always) to generate a total score (20–80), with higher scores indicating greater feelings of loneliness. Internal consistency for this study was 0.70. A cut-off value ≥ 50 was used to identify cases of moderately high loneliness [35].

### 3.6. Statistical Analysis 

Data were analyzed with the Statistical Package for the Social Sciences (SPSS) Version 22 and AMOS 22. Missing data were minimal across scales (<5%) and were imputed with mean replacement. According to the skewness (Sk) and kurtosis (Ku) values, the study variables can be considered almost normally distributed, with the highest skewness value being 0.92 and kurtosis 1.12. Descriptive statistics were calculated, and zero-order correlations were used to examine bivariate relations between the main study variables. 

The primary analysis involved path analysis, a simplified form of structural equation modeling without extraction of latent constructs, to explore whether ambivalence over emotional expression (the mediator) would be of importance in explaining the association between the severity of childhood trauma and self-discrepancy (the independent variables) and loneliness (the dependent variable). We controlled for types of childhood trauma as assessed by ITEC (experience of physical or sexual trauma and neglect or emotional abuse), but these factors were non-significant and were not further discussed.

Maximum likelihood estimation was used to estimate parameters. Model fit was assessed using three indices recommended by Hu and Bentler [36]: Root Mean Square Error of Approximation (RMSEA) and its 90% Confidence Interval (CI) (values close to 0.06 considered indicative of good fit and values up to 0.08 indicative of adequate fit); the Bentler-Bonett Normed Fit Index (NFI; values ≥ 0.90 indicative of adequate fit, and ≥0.95 indicative of good fit); and the Comparative Fit Index (CFI; values ≥ 0.90 indicative of adequate fit, and ≥0.95 indicative of good fit). The indirect and mediation effects were assessed using a bootstrap 95% CI sampled 1000 times, with statistical significance determined by the absence of zero within the lower and upper bounds of the CI [37]. In this study, the level of significance was set at 0.05. Based on simulations by Fritz and Mackinnon [38], a sample size of 148 would allow for the detection of mediated effects involving pathways sized between small and medium with 0.80 power. 

## 4. Results

### 4.1. Descriptive Statistics

Table 1 shows the results of the descriptive statistics (*N* = 133). The age among the patients ranged from 30 to 66 years, with a mean of 51.02 years (SD = 9.29). In terms of relationship status, 83.5% were married, 5.3% were single, and 11.3% were divorced or widowed. The highest educational attainment was reported as a diploma degree for 46.6% and a graduate or undergraduate degree for 27.1%. Most participants were unemployed (71.4 %) and had middle socioeconomic status (91.7%). 

Regarding childhood trauma, 28.6% (*n* = 38) of participants had experienced sexual or physical trauma, and 85.7% (*n* = 114) had experienced emotional trauma or neglect. Based on cut-off values, 40% (*n* = 54) reported childhood trauma of moderate severity, 18% (*n* = 24) reported self-discrepancy of moderate severity, and 24% (*n* = 32) reported emotional ambivalence of moderate severity. Lastly, 35% (*n* = 47) of the sample reported loneliness of moderately high severity. 

For the S-DS, patients were asked to generate 12 traits for the ideal and 12 traits for the ought self. The mean percentage of self-identification for positive ideal self traits was 83% (SD = 4.02), and positive ought self traits was 74% (SD = 5.55). In addition, the mean percentage of self-identification for negative ideal self traits was 77% (SD = 5.97) and negative ought self traits was 69% (SD = 5.24). According to a content analysis of these traits (see Table 2), patients with breast cancer wanted to be more spiritual, cheerful, humble, and compassionate, while they did not want to be frail, solitary, anxious, or depressed. Regarding the ought self, these patients stated that the important people in their lives would like them to be more tolerant, calm, optimistic, and spiritual, but not be tired, pessimistic, worried, or alone.

Table 3 shows descriptive statistics and zero-order correlations for the main study variables. The severity of childhood trauma was positively correlated with ambivalence over emotional expression (r = 0.15, *p* < 0.05) and loneliness (r = 0.26, *p* < 0.01). Furthermore, ambivalence over emotional expression was significantly correlated with loneliness (r = 0.21, *p* < 0.05). In addition, a significant positive relationship between self-discrepancy and ambivalence over emotional expression (r = 0.24, *p* < 0.01) was observed. Lastly, the relationship between self-discrepancy and loneliness was significant (r = 0.30, *p* < 0.01).

### 4.2. Evaluation of the Structural Model

We investigated the specific paths between the severity of childhood trauma, self-discrepancy, ambivalence over emotional expression, and loneliness. First, the full mediation model was tested, wherein it was examined if ambivalence over emotional expression fully mediated the relationships between traumatic childhood experiences and self-discrepancy with loneliness as the dependent variable. That is, the full mediation model did not have any direct pathways from CT and S-DS to loneliness. Therefore, the full mediation model did not demonstrate an acceptable fit: CFI = 0.71; NFI = 0.73; RMSEA = 0.13 (90% CI = 0.04 to 0.19). 

**Figure 1:**
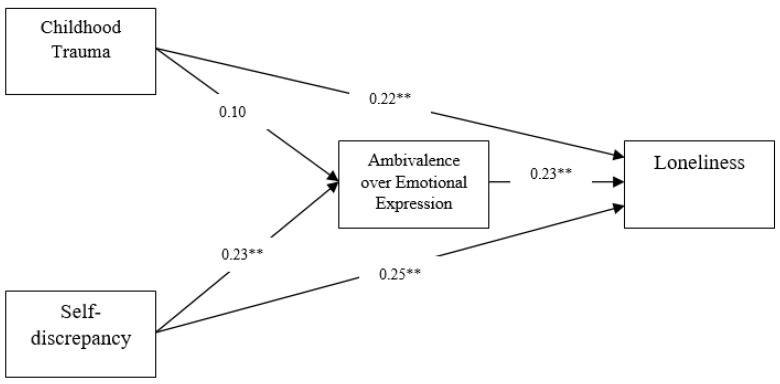
Ambivalence over emotional expression as a partial mediator between childhood trauma/self-discrepancy and loneliness. Note. Standardized parameter estimates are provided. Covariates not shown. ** *p* < 0.01.

We next tested a partial mediation model that allowed for direct paths from traumatic childhood experiences and self-discrepancy to loneliness, and compared this to the full mediation model. The partial mediation model (see Table 4) showed an acceptable fit (CFI = 0.96; NFI = 0.95; RMSEA = 0.08 (90% CI = 0.03 to 0.12)) and was considered the most appropriate model (see Figure 1). 

As shown in Table 4, the severity of childhood trauma was unrelated to ambivalence over emotional expression (*β* = 0.10, *p* > 0.05). However, self-discrepancy was positively related to ambivalence over emotional expression (*β* = 0.23, *p* < 0.01). The pathway between ambivalence over emotional expression and loneliness was significant (*β* = 0.23, *p* < 0.01). Both traumatic childhood experiences (*β* = 0.22, *p* < 0.01) and self-discrepancy (*β* = 0.25, *p* < 0.01) still significantly predicted loneliness even with ambivalence over emotional expression in the model. 

### 4.3. Tests of Mediation Effects 

Indirect effects in the model were tested with a bootstrap procedure [37]. Examination of the 95% bias-corrected CI from 1000 bootstrap samples revealed that the mediation effect of traumatic childhood experiences (B = 0.01, *p* = 0.08, 95% CI: −0.003 to 0.00) on loneliness through emotional ambivalence was not significant (see Table 4). However, there was a significant mediation effect from self-discrepancy (B = 0.11, *p* = 0.03, 95% CI: 0.003 to 0.16) to loneliness via emotional ambivalence (see Table 4). Ambivalence over emotional expression was a partial mediator, controlling for the type of childhood trauma. 

## 5. Discussion

In this study, we investigated the factors relating to the experience of loneliness in patients with breast cancer who had experienced early childhood trauma. Based on an initial screening of 408 women with breast cancer living in Tabriz, Iran, we found that 42% (*n* = 172) had experienced some form of childhood abuse or neglect. In the final sample of 133 participants with breast cancer and early childhood trauma, 29% reported the experience of physical or sexual abuse, and 86% reported the experience of neglect or emotional abuse. 40% of participants reported childhood trauma of moderate severity. These rates may have been influenced by historical factors associated with exposure to war and conflict in Iran during early development [39].

In this sample of childhood trauma survivors, the experience of loneliness was substantial, with a third of participants reporting moderate to severe loneliness. In addition, we found that the severity of childhood trauma was directly associated with the experience of loneliness, independent of effects related to emotional ambivalence about the sharing of feelings with others and independent of negative changes in self-concept in the context of cancer. Although an association between traumatic childhood experiences and feelings of loneliness in adulthood has been demonstrated [40], this association has not been investigated in breast cancer. 

Regarding self-discrepancy, participants showed concern with spirituality, remaining tolerant and patient in the face of illness, and limiting the experience of frailty, weakness, and negative emotions. Greater self-discrepancy was associated with more ambivalence about emotional expression and loneliness, both directly and indirectly. Negative changes in self-concept provoked by the illness experience may create a dilemma for patients who can feel an increased need to express powerful feelings that are difficult to confront and difficult for others to hear [41]. The inability or lack of opportunity to share emotional experiences may lead to depression and anxiety [28]. 

## 6. Clinical Implications 

The diagnosis of cancer can reawaken dormant feelings and emotions caused by childhood trauma, affecting adaptation to cancer later in life [3,6], which may obligate oncology professionals active in the field not to limit themselves to the physical treatment of patients but also attend to emotional needs with the assistance of psychologists and psychiatrists. However, psychosocial oncology is still in its early development in Iran, and the integration of psychological medicine into oncology practice can be strengthened. 

Routine systematic psychosocial screening for childhood trauma and emotional difficulties may be recommended in newly diagnosed breast cancer patients [42]. Oncology professionals may benefit from psychoeducation about the late effects of childhood trauma in the context of medical illness and training concerning trauma-informed approaches to care [43]. Patients with childhood trauma should have the option to speak with a psychosocial expert concerning their history if they want to. 

Referral to psychology and psychiatry should occur for individuals identified by oncology staff as adapting poorly to disease, as indicated by moderate to severe loneliness and emotional disturbances. Where childhood trauma is implicated, Dialectical Behavior Therapy (DBT) and Cognitive Processing Therapy (CPT) have been found to be effective in adult women experiencing complex traumatic stress associated with childhood abuse [44]. Emotionally focused therapies [45], which can include music and art-based approaches to improve emotional expression [46], may benefit individuals struggling with emotional ambivalence. Individuals with negative self-discrepant views may also benefit from a reflective emotional space to process experiences and to promote cognitive reframing and acceptance of change. Effective treatments may require an understanding of cultural and family dynamics. Complex case management should consider a multidisciplinary approach that may evolve over time to become interdisciplinary. 

## 7. Limitations and Future Directions

Several limitations warrant consideration. First, the sample was of relatively small size, composed only of women, and was restricted to hospitals in Tabriz. Second, there was no assessment of patients without childhood trauma to offer a control group for comparison. Third, there may be recall bias of childhood traumatic experiences and other self-report issues with measures. Finally, findings were based on cross-sectional data, limiting conclusions about causal effects. Future work may be improved with the multidimensional modeling of latent constructs and the development of a more comprehensive theoretical framework. Although this study has focused on women with breast cancer, the effects of childhood trauma and loneliness on quality of life and treatment outcomes deserve wider examination in cancer populations more generally and consideration of gender. 

Due to our clinical context, this study has focused on negative adaptations to CT and cancer. Post-traumatic growth refers to positive psychological changes that can follow the experience of life adversity—growth in response to cancer is possible, although its relationship to stress may not be clear-cut according to a recent review [47]. The relationship may not be correlational but rather one of necessity vs. sufficiency. Some stress and adversity is needed to stimulate growth, but if the challenge exceeds the person’s zone of proximal development, then they will be weakened by the experience rather than made more resilient. Effective therapeutic support may extend the individual’s psychosocial capacity to cope with adversity. Future work could assess positive psychological changes following a supportive intervention, in addition to traditional reductions in negative affect. 

## 8. Conclusions

Childhood trauma may be prevalent in women with breast cancer and may continue to exert negative effects on the experience of social connection throughout the illness trajectory. Therefore, it is recommended that healthcare professionals consider an assessment of childhood adversity as part of routine psychosocial oncology care and that a trauma-informed treatment approach may improve the healing process, social connectedness, and quality of life of patients with breast cancer affected by these childhood experiences.

## Figures and Tables

**Table 1:** Sample characteristics (*N* = 133).

Variable	*N*	%
Marital status		
Married	111	83.5
Single	7	5.3
Widowed/Divorced	15	11.3
Education		
Elementary	35	26.3
Diploma	62	46.6
Undergraduate or Graduate	36	27.1
Socioeconomic status		
High	8	6.0
Middle	122	91.7
Low	3	2.3
Employment status		
Employed	17	12.8
Unemployed	95	71.4
Retired	21	15.8
	M	SD
Age	51.02	9.29

**Table 2:** Content analysis of S-DS attributes.

Type		Attributes	%
Ideal self	Positive	Spiritual	11.1
		Cheerful, optimist, happy	7.8
		Humble, modest	7.3
		Grateful	6.7
		Sympathetic, compassionate	6.3
		Kind, friendliness, cordial, warm	5.7
		Agreeable, conformist	5.7
		Careful, cautious	5.2
		Calm, gentle	5.2
		Honest	3.3
		Wise, calculating	3.2
		Enthusiastic	2.3
		Reasonable, logic	2.3
		Clever, intellectual	2.2
		Adventurous	2.1
		Hardworking, achievement-focused, moral, serious	1.9
		Helpful	1.5
		Active, assertive	1.3
		Liberal	1.1
		Other	17.8
		Frail, weak, tired	13.1
	Negative	Solitary, shy, alone	9.9
		Anxious, worried, neurotic	9.7
		Depressed, painful, pessimist	9.1
		Disagreeable, disinterested	8.7
		Childish, selfish	5.1
		Aggressive	4.4
		Sentimental, emotional, sensitive	3.6
		Arrogant, proud, stubborn	2.6
		Hard	2.3
		Envious	2.3
		Lazy	2.2
		Obedient, submissive	2.1
		Disorderly, impulsive	1.3
		Other	23.6
		Tolerant, patient	14.1
Ought self	Positive	Calm, gentle	10.1
		Cheerful, optimist, happy	9.3
		Spiritual	8.1
		Wise, calculating	7.3
		Dutiful	7.1
		Humble, modest	5.5
		Honest	3.2
		Careful, cautious	2.4
		Sympathetic, compassionate	2.1
		Polite	2.1
		Kind, friendliness, cordial, warm	2.1
		Agreeable, conformist	2.1
		Dependable, reliable	1.7
		Altruist	1.3
		Other	21.5
		Frail, weak, tired	13.1
		Depressed, painful, pessimist	12.1
	Negative	Anxious, worried, neurotic	11.5
		Solitary, shy, alone	10.2
		Obedient, submissive	8.6
		Sentimental, emotional, sensitive	7.2
		Lazy	4.8
		Insensitive	3.9
		Obstinate	3.1
		Unpredictable	2.5
		Stingy, spiteful	2.3
		Credulous	1.8
		Other	18.9

**Table 3:** Descriptive statistics and zero-order correlations for study variables.

	Predictors		Mediator	Outcome
	1	2	3	4
1. CASRS	1			
2. S-DS	0.07	1		
3. AEE	0.15 *	0.24 **	1	
4. UCLA LS3	0.26 **	0.30 **	0.21 *	1
Mean	22.301	12.23	71.04	33.95
SD	13.87	5.79	13.53	9.46
Min	4	4	40	20
Max	89	28	113	70
Cronbach’s Alpha	0.83	0.84	0.72	0.70

* *p* < 0.05. ** *p* < 0.01. Note: CASRS, Child Abuse Self-Report Scale; S-DS, Self-Discrepancies Scale; AEE, Ambivalence over Emotional Expression; UCLA LS3, The University of California, Los Angeles (UCLA), Loneliness Scale-Version 3.

**Table 4:** Results and fit indices of the partial mediation model.

Path	Childhood Trauma to		Self-Discrepancy to		Ambivalence to	Total Effect: Trauma to Loneliness	Total Indirect: Trauma to Loneliness	Mediation Effect: Trauma →Ambivalence → Loneliness	Total Effect: Self-Discrepancy to Loneliness	Total Indirect: Self-Discrepancy to Loneliness	Mediation Effect: Self-Discrepancy → Ambivalence → Loneliness
	Ambivalence	Loneliness	Ambivalence	Loneliness	Loneliness						
B (SE)	0.10 (0.08)	0.15 (0.05)	0.53 (0.18)	0.41 (0.13)	0.15 (0.05)	0.16 (0.10)	0.02 (0.01)	0.01 (0.01)	0.44 (0.15)	0.10 (0.05)	0.11 (0.04)
β	0.10	0.22 **	0.23 **	0.25 **	0.23 **	0.24 **	0.02	0.015	0.28 **	0.06 *	0.07 *
95% CI	[−0.06 to 0.24]	[0.007 to 0.41]	[0.05 to 0.38]	[0.09 to 0.38]	[0.04 to 0.31]	[0.03 to 0.43]	[−0.006 to 0.08]	[−0.003 to 0.00]	[0.09 to 0.41]	[0.004 to 0.14]	[0.003 to 0.16]
		Fit index									
	CFI	NFI	RMSEA								
Full mediation	0.71	0.73	0.13								
Partial mediation	0.96	0.95	0.08								

Note that the total indirect effect refers to an estimate of the indirect effect without the presence of c’ (i.e., the direct pathway) in the model. The mediation effects retain c’ in the models. * *p* < 0.05. **p < 0.01.

## Data Availability

There is no plan to share data publicly. Data requests may be considered on a case-by-case basis.
